# Modern Aspects of the Structural and Functional Organization of the DNA Mismatch Repair System

**Published:** 2013

**Authors:** S. A. Perevoztchikova, E. A. Romanova, T. S. Oretskaya, P. Friedhoff, E. A. Kubareva

**Affiliations:** Belozersky Institute of Physico-Chemical Biology, Lomonosov Moscow State University, Leninskie Gory, 1, bld. 40, Moscow, Russia, 119991; Chemistry Department, Lomonosov Moscow State University, Leninskie Gory, 1, bld. 3, Moscow, Russia, 119991; Institute of Biochemistry, FB 08, Justus Liebig University, Heinrich-Buff-Ring 58, D-35392 Giessen, Germany

**Keywords:** DNA mismatch repair system, structure of proteins, protein-protein and protein-DNA interactions, MutS, MutL, MutH

## Abstract

This review is focused on the general aspects of the DNA mismatch repair (MMR) process. The key proteins of the DNA mismatch repair system are MutS and MutL. To date, their main structural and functional characteristics have been thoroughly studied. However, different opinions exist about the initial stages of the mismatch repair process with the participation of these proteins. This review aims to summarize the data on the relationship between the two MutS functions, ATPase and DNA-binding, and to systematize various models of coordination between the mismatch site and the strand discrimination site in DNA. To test these models, novel techniques for the trapping of short-living complexes that appear at different MMR stages are to be developed.

## INTRODUCTION

The genome is the primary repository of the information necessary for the survival of any organism. Replication of the genetic material in unaltered form during the somatic and generative cell division is the most important condition for the existence and maintenance of the viability of organisms. A single nucleotide substitution in a single gene can lead to developmental disorders or even to a lethal outcome if the former occurs in germ cells [1] or to carcinogenesis if mutations occur in somatic cells [2]. Errors take place during replication regardless of the correcting activity of DNA polymerases. It is estimated that on average one nucleotide substitution occurs per 10^8^–10^10^ nucleotides during the replication of DNA by bacterial DNA polymerase [3]. Not all eukaryotic DNA polymerases possess a 3'→5'-exonuclease activity, which leads to a large error rate [4], and, therefore, the need for systems repairing the incorrectly inserted nucleotides that could prevent the occurrence of mutations is evident. Currently, from five to nine systems involved in the damage repair can be identified, amongst which the mechanisms of direct repair, excision repair, post-replicative and SOS-repair are being extensively investigated [5, 6]. A DNA mismatch repair (MMR) also performs an important role in the maintenance of the genome's stability. The need for research concerning maintenance of the genetic stability is supported by the large number of experimental and review articles on the subject. The most important achievement is discussed every year in the penultimate issue of the journal “Science.” In 1994, the topic was DNA repair [7]. The first issue of “Biochemistry” (Moscow) in 2011 [8] was devoted to the mechanisms of DNA damage repair systems. In the present review we consider the DNA mismatch repair system. Over the past decade and a half, a significant number of review papers have been dedicated to the repair of mismatches [9 – 17]. We considered the experimental data, including those obtained during the last 5–6 years, and an attempt to systematize the understanding of the mechanisms by which the MMR system functions was made.

## THE ROLE OF THE MMR SYSTEM IN BIOLOGICAL PROCESSES

Mismatches are considered to be any nucleotide pairs other than G/C and A/T. Their occurrence is caused by erroneous insertion of nucleotides by DNA polymerase during the copying of the template strand, as well as the influence of mutagenic factors (including free radicals and ionizing radiation). Insertion of modified nucleotides carried out by DNA polymerase or an unmodified nucleotide opposite the damaged base in the template strand is feasible [5, 18].

Another common error of the replication system is short insertion-deletion loops (IDL), which also occur during the formation of duplexes in the course of homologous recombination [19, 20]. The damages mentioned above are recognized and restored by the mismatch repair system (MMR), thereby reducing the likelihood of emergence of mutations by a factor of 50–1,000 [21, 22]. The MMR system is also involved in DNA restoration after the occurrence of certain chemical modifications. Repair of the following modifications has been demonstrated: O6-methylguanosine [23, 24], 8-oxoguanosine [25, 26], adducts formed during exposure of carcinogens on DNA [27], photo-induced compounds [28 – 30], and products of the reaction of DNA with cisplatin derivatives [31].

The role of the MMR system is not limited to the repair of the above-listed DNA lesions. The proteins of this system are involved in cell cycle regulation. In particular, during the G2 phase the DNA damage signal transmitted by the MutS protein triggers a cascade of processes that cause programmed cell death (apoptosis) [15, 32, 33]. Abnormality in this function leads to enhanced cell survival resulting in carcinogenesis, as well as resistance of these cells to chemotherapy [13, 25]. Likewise, defects in the mismatch repair system in prokaryotes lead to an increased rate of mutagenesis and to interspecies gene transfer, which can ensure adaptability of the bacteria to stressful conditions and to drug resistance [34].

The MMR system is vital for maintaining the length of microsatellite repeats, i.e. short repetitive DNA [13, 35, 36]. Replication of the repeated segments often leads to errors attributed to the slippage of the DNA polymerase to an analogous sequence. As most of the burden of the repair of these lesions lies with the MMR system, microsatellite instability is used as a biomarker for the abnormalities of the functioning of the proteins of this repair system. Dysfunctions within the MMR system result in various DNA rearrangements and telomerase- independent telomere lengthening [37, 38].

MMR system proteins are also important for the prevention of recombination between similar, but not identical, DNA sequences, as well as for chromosome pairing during meiosis and the segregation of chromosomes [39]. In somatic cells the MMR is involved in hypermutation during the formation of the repertoire of immunoglobulins in B lymphocytes [40, 41]. The wide variety of biological functions of the mismatch repair system draws interest regarding the details of its mechanisms.

The mismatch repair system has been discovered in all kingdoms of living organisms; its key proteins – MutS and MutL – are highly conserved across species, from bacteria to higher eukaryotes [42]. Given the structural similarity of the proteins, it is assumed that the principles of the mismatch repair mechanisms are similar in all organisms. Defects in the MMR system proteins in humans lead to the emergence of tumors, including malignant ones. The Lynch syndrome or hereditary nonpolyposis colon cancer (HNPCC ) is the most common amongst them. Mortality rates associated with the latter ranks third amongst cancers [43 – 46]. Mutations in the genes encoding the proteins of the MMR system are identified in 85% of hereditary nonpolyposis colon cancer cases [44] and in 15–25% of cases of sporadic tumors of various tissues [47]. Detection of abnormalities in the MMR system plays an important role in the diagnosis of tumors [48]. The existence of a link between human cancers and the MMR determines the relevance of investigations of the DNA mismatch repair system.

In 1989 the MMR process was reconstituted *in vitro* using purified components [49], and currently the general scheme of how the MMR system works is well understood. However, many questions remain to be resolved in order to create an adequate model of the MMR process. The general views on the mechanism of MMR are presented below.

## OVERVIEW OF THE MECHANISM AND ORDER OF EVENTS IN THE MMR PROCESS

The key proteins of the MMR system are MutS and MutL. The genes encoding these proteins were originally discovered in *Streptococcus pneumoniae *(*hexA* and *hexB *genes) [50]. Somewhat later, homologous genes were discovered in *Escherichia coli *(*mutS *gene,* hexA *homologue, and *mutL *gene, *hexB *homologue), as well as *mutH *and *mutU *genes [51]. MMR system proteins were named Mut (short for mutagenic) as their dysfunction leads to hypermutability in microorganisms. Genes encoding proteins that are homologous to MutS and MutL have been discovered in the majority of sequenced genomes. The names of MutS and MutL homologues are formed using the abbreviations MSH (from MutS homologue) and MLH (from MutL homologue), respectively.

MMR is a multicomponent system. Its function requires the coordinated action of over 10 proteins [52]. *Table 1 *shows the key proteins of the MMR system in *E. coli* and humans, and their functions are compared.

**Fig. 1:**
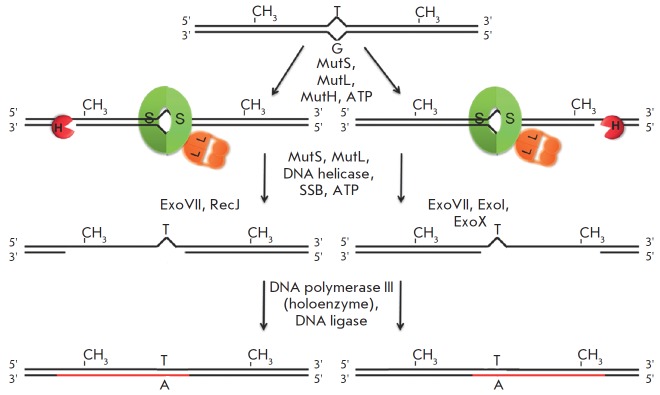
The scheme showing the DNA mismatch repair process in *E. coli*

The general scheme of the mismatch repair in *E. coli* is shown in *Fig. 1*. MutS acts as a sensor scanning the DNA searching the mismatches: G/T, C/T, A/C, A/G, G/G, A/A, T/T (all but the C/C), and small insertiondeletion loops (IDL) [14]. Over the past years, it has been demonstrated that MutS also stimulates the cellular response to damaging agents in mammals such as cisplatin, ionizing radiation, antimetabolites, ultraviolet radiation, and alkylating and intercalating agents [24 – 31, 53, 54]. MutS forms specific contacts with a mismatch in the so-called initial recognition complex (IRC ) characterized by bending of the DNA by 60° [13, 55]. The MutS protein then interacts with the MutL protein, forming a ternary complex which acts as a coordinator of subsequent processes, including distinguishing between the parent and the daughter (i.e., containing the error) DNA strands. MutS and MutL are ATPases: their functioning requires the presence of ADP and ATP [13, 14].

**Table 1:** Key proteins of the E.coli and human mismatch repair systems

*E. coli*	Function	Homologue in human cells	Function
MutS (homodimer)	Recognition of mismatches	MSH2–MSH6 (MutSα)	Repair of mismatch and insertion-deletion loops consisting of 1-2 nucleotides
		MSH2–MSH3 (MutSβ)	Repair of insertion-deletion loops consisting of 2 or more nucleotides
		MSH4–MSH5	Participation in the meiotic recombination and in the order of switching of immunoglobulin synthesis
MutL (homodimer)	Coordination of the MMR processes after recognition of a mismatch and before reparative biosynthesis of DNA	MLH1–PMS2 (MutLα)	As per MutL from *E. coli*
		MLH1–PMS1 (MutLβ)	Suppression of insertion-deletion mutagenesis in yeast homologues; the function of the human homologue in the MMR is unclear
		MLH1–MLH3 (MutLγ)	Suppression of insertion-deletion mutagenesis; participation in meiotic recombination
MutH	Recognition of 5'-Gm^6^ATC -3'/ 3'-CT AG↓-5' and hydrolysis of the daughter unmethylated DNA strand	Not identified	

The absence of methylation in the newly synthesized strand plays an important role in distinguishing between the parental and the daughter DNA strands in enterobacteria. Hence, the MMR system in such bacteria is called a methyl-directed mismatch repair system. This relationship was discovered by Meselson *et al*. [56, 57], who investigated the repair of bacteriophage λ carrying one or several mismatches after its transfecting into *E. coli *strains. It was found that the repair of closely positioned mismatches occurs in the same DNA strand [56]. Involvement of the MutH protein, a DNA nicking enzyme responsible for recognizing the hemimethylated sequence 5'-Gm^6^ATC -3'/3'-CT AG↓-5' (where m^6^A is N6-methyl-2'-deoxyadenosine; the arrow indicates the position of hydrolysis), is important during the selection of the DNA strand in which to introduce a break and to start the subsequent excision repair. The emergence of MutH recognition sites is associated with the action of cellular Dam-methyltransferase. Before DNA replication is initiated, the adenosine residues of both strands of the 5'-GATC -3' sequences are methylated within the cell. However, for a certain period of time after replication, the cell contains a pool of DNA in which only one of the two strands is methylated [58]. MutH catalyzes the single-stranded break in the unmethylated, i.e. newly synthesized DNA strand [16, 59]. Fully methylated DNA in *E. coli *cells does not undergo a repair process [60], and in the absence of methylation (*dam- *strains) distinguishing between parent and daughter strands is impossible, which may lead to double-stranded DNA breaks. Therefore, *E. coli *strains with insufficiently and excessively active Dam methyltransferase demonstrate an increased rate of mutagenisis [61, 62]. The catalytic function of MutH is stimulated by a ternary complex consisting of the MutS and MutL proteins and DNA containing a mismatch. Typically, MutH bound to its recognition site and located in the nearest possible position to the mismatch on either side of the DNA relative to the mismatch is activated. The distance between the mismatch and the site of strand discrimination can reach 2,000 bp [14, 63].

Mismatch repair is independent of DNA methylation in the cells of most other organisms. The question of how the repair system detects the daughter strand, i.e. the strand containing an error, remains open to discussion. Introduction of a break into the DNA in such organisms is attributed to MutL homologues in which an endonuclease motif was discovered [64]; however, this fact has not been confirmed experimentally. Another assumption is that single-stranded breaks occurring in the course of DNA replication may serve as signals of a newly synthesized DNA strand: from the 3'-end of the leading strand and the 3'- and 5'-ends of the lagging strand [65].

The single-stranded break serves as a signal for excision steps of the repair process in which a fragment of a DNA strand containing a mismatch is removed. The DNA helicase UvrD binds to the nick and unwinds the DNA until a non-canonical base pair is reached. It has been shown that the action of a DNA helicase is stimulated by the ternary MutS-MutL-DNA complex and directed towards the mismatch [66 – 68]. The latter indirectly indicates the ability of a ternary complex to coordinate the recognition of a mismatch and the subsequent occurrence of excision repair. The released single-stranded DNA is hydrolyzed by a specific set of exonucleases depending on whether the 5'- or 3'-end is accessible [69, 70]. The single-strand binding protein (SSB) interacts with the parent DNA strand covering its entire surface and preventing degradation [71, 72]. The single-stranded gap is rebuilt by DNA polymerase III. DNA ligase restores the integrity of the corrected strand.

## MutS AS A KEY PROTEIN OF THE MMR SYSTEM

**Fig. 2:**
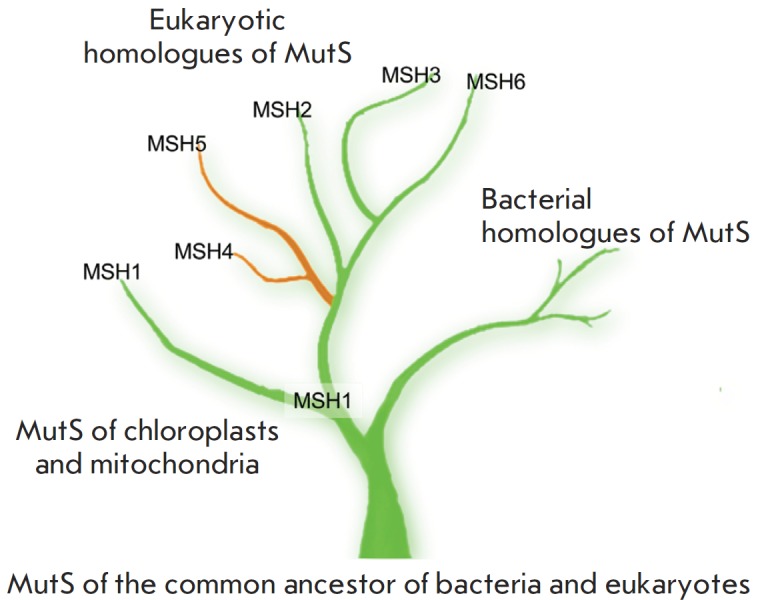
The phylogenetic tree of MutS family proteins. Green branches represent MutS homologues involved in the maintenance of genetic material stability during vegetative cell division; the brown branches – during meiotic DNA recombination

A substantial amount of structural and biochemical data regarding the protein MutS and its homologues has been accumulated. The MutS protein from *E. coli *is a polypeptide with a molecular weight of 95 kDa. The MutS quaternary structure in the solution is an equilibrium mixture of dimers and tetramers [73] formed by the equivalent subunits (with regards to the primary structure). In eukaryotes, MutS homologues forms dimers from two different subunits. Six human homologues of MutS (MSH1–MSH6) have been identified. Heterodimers, known as MutSα (MSH2–MSH6) and MutSβ (MSH2–MSH3), together perform the functions of the bacterial MutS protein, ensuring accuracy in mitotic replication (*Table 1*). MSH1 supports genetic stability in the mitochondria of eukaryotes [20]. The MSH4–MSH5 heterodimer is involved in the resolution of Holliday junctions during meiosis [74 – 76] and does not participate in the repair of replication errors. A bioinformatics analysis enables to construct a phylogenetic tree that reflects the functional specialization of MutS homologues [77] (*Fig. 2*).

## Structure of the MutS protein from E. coli and functions of its individual domains

An important milestone in investigations of the MutS protein was the elucidation of its crystal structure. In 2000, the crystal structures of MutS–DNA complexes from *E. coli *[55] and *Thermus aquaticus *[78] containing a non-canonical pair were solved. Crystals of the MutS proteins and their mutant forms in complexes with DNA containing various mismatches were obtained later [79-83]. From amongst the eukaryotic MutS homologues, the structures of human MSHα and MSHβ have been elucidated. To date the structures of over 20 MutS–DNA complexes [55, 78, 79, 81-86] have been determined; the corresponding data are openly available in the Protein Data Bank (PDB) (*Table 2*).

It should be noted that the structures of all MutSDNA complexes obtained by X-ray diffraction analysis (XRD) are very similar. They represent the initial recognition complex (IRC ) of the MutS with DNA containing a mismatch. In these structures the MutS protein forms specific contacts with a mismatch and is bound to a cofactor, ADP. The only structure of the MutS-DNA complex containing a G/T-mismatch and two molecules of ATP (code PDB 1W7A) was obtained by soaking of the crystals in an ATP solution. In this case the molecules remained firmly fixed in the crystal lattice, which prevented significant conformational rearrangements of the complex [85]. Data regarding the structure of the protein at the stage of scanning of the DNA in search of a mismatch or during the stage of signal transduction to other components of the MMR repair system cannot be obtained, which is attributed to the high dynamics of MutS-DNA complexes during these stages.

**Fig. 3:**
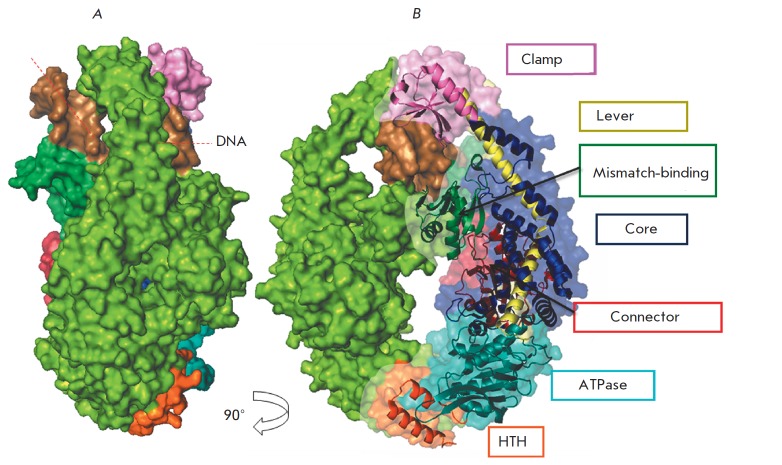
The overall structure of the MutS from *E. coli *in complex with DNA containing a G/T-mismatch*. *Lateral view (**A**) and frontal view (**B**) are presented. DNA is colored in brown, MutS subunit 2 – in green. The domains of DNA-binding subunit 1 are shown in picture **B**: the mismatch-binding domain (aa 2–115) is colored in dark green; the connector domain (aa 116–266) – in red; the core domain (aa 267–443) – in blue; the lever domain (aa 504–567) – in yellow; the clamp domain (aa 444–503) – in pink; the ATPase domain (aa 568–765) – in cyan; and the HTH domain (aa 766–800) – in orange. The DNA kink is marked by a red dashed line (PDB code 1E3M)

The primary structure of MutS is highly conserved across all living organisms. The secondary and tertiary structures of this protein in different organisms are highly conserved. In complex with the DNA, the protein is a dimer of elongated shape with two channels (each approximately 100 A^°^ in length). Its shape resembles the Greek letter θ [87] (*Fig. 3A, B*). While the duplex with a mismatch is located in the larger channel, the function of the second channel remains unknown. However, its size and charge lead to conclude that it is capable of forming contacts with DNA [82].

XRD was used to obtain a high-resolution structure of the protein (less than 2 A^°^). Attempts to characterize the structures of four regions (aa 2–13, 57–66, 95– 107 and 659–668) in the DNA-free protein (PDB-code 1EWR) have failed, indicating the conformational mobility of the protein in the absence of DNA. The positions of all amino acids, except for the loop formed by the aa 659–668, have been determined in MutS–DNA complexes containing a mismatch [55].

Each MutS monomer has seven structural domains (*Fig. 3B*). The N-terminal domain is a mismatch-binding (aa 2–115) one. This domain is formed by a mixed s-sheet layer consisting of five strands and surrounding the latter three α-helices. The following adjacent domain, which is a connector domain (aa 116–266), is primarily composed of parallel s-strands surrounded by four α-helices. The core domain (aa 267–443 and 540–567) comprises two bundles of α-helices. The lever domain (aa 504–567) consists of two α-helices protruding out of the core domain and surrounding the DNA but lacking direct contact with the latter. An important feature of the structures of prokaryotic and eukaryotic MutS homologues is a long α-helix consisting of 60 aa which connects the core domain to the clamp domain. The helix is likely to be involved in the signal transduction between the ATPase and the DNA-binding domains [86]. The clamp domain (aa 444–503) is an insertion into the upper part of the lever domain. It is formed by four antiparallel s-strands. The nucleotidebinding (ATPase) domain (aa 568–765) and the HTH (helix-turn-helix) domain (aa 766–800) are located in the C-terminal region of the protein.

**Table 2:** Crystal structures of the MutS protein

Organism	DNA^1^	ATP or ADP	Resolution, A^°^	PDB code	Reference	Substitution, aa
*E. coli^2^*	G/T	ADP	2.20	1E3M	[55]	-
	«	«	2.10	1WB9	[84]	E38T
	«	«	2.50	1WBB	[84]	E38A
	«	«	2.40	1WBD	[84]	E38Q
	«	«	2.20	3K0S	[83]	D693N
	«	ADP (2 molecules)	2.60	1NG9	[79]	R697A
	«	ADP (2 molecules)	2.27	1W7A	[85]	-
	A/A	ADP	2.40	1OH6	[81]	-
	A/A	«	3.40	2WTU	[83]	-
	G/G	«	2.50	1OH7	[81]	-
	C/A	«	2.90	1OH5	[81]	-
	extra T	«	2.90	1OH8	[81]	-
*Thermus* *aquaticus^3^*	-	-	3.19	1EWR	[78]	-
	extra T	-	2.20	1EWQ	[78]	-
	extra T	ADP (2 molecules)	2.70	1FW6	[79]	-
	extra T	ADP (2 molecules)·BeF^3^	3.11	1NNE	[82]	-
Human (MSHα)	G/T	ADP	3.30	2O8E	[86]	-
	G/T	ADP (2 molecules)	2.75	2O8B	«	-
	G/dU	ADP	3.00	2O8D	«	-
	m^6^G/T ^4^	«	3.37	2O8C	«	-
	extra T	«	3.25	2O8F	«	-
Human (MSHβ)	loop 4 n.r.^5^	«	3.09	3THW	-	-
	loop 3 n.r.	«	2.70	3THX	-	-
	loop 2 n.r.	«	2.89	3THY	-	-
	loop 6 n.r.	«	4.30	3THZ	-	-

1 Non-canonical pair of nucleotides in the DNA duplex used for crystallization is shown. 2 In the case of MutS from E. coli deletion variants containing aa 1–800 were used. 3 In the case of MutS from T. aquaticus deletion variants containing aa 1–782 were used. 4 m^6^G – O6-methyl-2’-deoxyguanosine. 5 n.r. – nucleotide residues.

Within the structure of the complex of MutS with the DNA containing a mismatch the protein is a homodimer arranged asymmetrically. The subunit forming specific contacts with the mismatch is hereinafter referred to as subunit 1 (in *Fig. 3*its domains are shown in different colors). The second subunit that forms contacts only with the DNA sugar-phosphate backbone is hereinafter referred to as subunit 2 (in *Fig. 3*it is shown in green). The protein surrounds the DNA in the location of a mismatch, covering an area comprising 24–28 bp [88]. The MutS protein covers the DNA in the form of a clamp. The binding of the protein to the DNA requires the clamp to “open up.” It is believed that the opening of the clamp is promoted by the flexible structure of the upper part of the domain that contains a large percentage of loops [89]. The flexibility of the DNA-binding domains is confirmed by the fact that the former are not structured in the crystals of the DNAfree MutS [78].

In a specific complex with MutS, the DNA is bent by 60° [78, 79] (*Fig. 3*). A mismatch is located at the apex of the corner. Bending results in expansion of the minor groove of the DNA in a manner that its width becomes approximately equal to the major groove width. Within the specific complex, the aa of both MutS subunits interact with the DNA; however, binding is asymmetrical – each subunit forms multiple contacts; however, they are all different. The total surface area of the DNA-protein contacts is ~ 1850 A^°2^ [81]. The majority of the contacts between the protein and the DNA are hydrophilic (aa interact with the sugarphosphate backbone of the DNA) and do not depend on the nucleotide sequence. Hence, MutS can function in various nucleotide contexts. Only amino acids from the subunit 1 (Phe-X-Glu motif) form specific contacts with a mismatch [86]. With respect to eukaryotic homologues, this motif is present in MSH6 but absent in MSH2 and MSH3. Even prior to the availability of XRD results, it was established that Phe36 (numbering for MutS from *E. coli*) performs an important role in the binding of MutS to DNA. Replacement of Phe36 with Ala disrupts the ability of MutS to engage in a specific interaction with DNA [90]. Perhaps, Phe36 is important in the search for a mismatch. According to XRD data, phenylalanine from the Phe-X-Glu motif is involved in the stacking with one of the heterocyclic bases of a mismatch on the minor groove side of the DNA [55, 78]. In the specific binding of MutS to a DNA mismatch, an important role is also performed by Glu38 (numbering for MutS from *E. coli*), which, similar to Phe36, forms contacts with the same heterocyclic base. The results of this interaction include the formation of a hydrogen bond between the carbonyl oxygen of Glu38 and the base nitrogen atom. Glu38 forms a hydrogen bond with the N3-atom of the T in the structures of MutS with a duplex containing a G/T pair or an unpaired nucleotide T. Glu38 forms a hydrogen bond with the N7-atom of the purine during the interaction of MutS with duplexes containing C/A and A/A pairs; an analogous contact is also formed with a non-canonical G/G pair [81]. Specific contacts determine the direction of the bend in the DNA. It was demonstrated that the replacement of a conserved residue of Glu38 with glutamine completely disrupts the ability of the protein to distinguish between canonical and mismatch-containing duplexes [91].

Unfortunately, little is known about the structure of the non-specific complex of MutS with the canonical DNA (homoduplex) as crystals of MutS with this DNA fragment could not be obtained. Sixma [89] suggests that the protein searches for a mismatch using the bind-release mechanism attempting to insert Phe36 into the “stack” of bases at each stage and, as a result, kink the DNA. The mismatch does not typically distort the structure of a DNA duplex [92, 93] but destabilizes it [94]. Natrajan *et al*. [81] suggest that MutS is able to detect these local weakening in the structure of the DNA. Atomic force microscopy demonstrated that DNA of non-canonical content in complex with MutS can be found in one of two conformations: bent or unbent [95]. It is believed that in the search for a mismatch MutS continuously bends and straightens the DNA. Detection of a mismatch leads to ATP-dependent rearrangements of MutS domains and the formation of the activated DNA-protein complex.

**Fig. 4:**
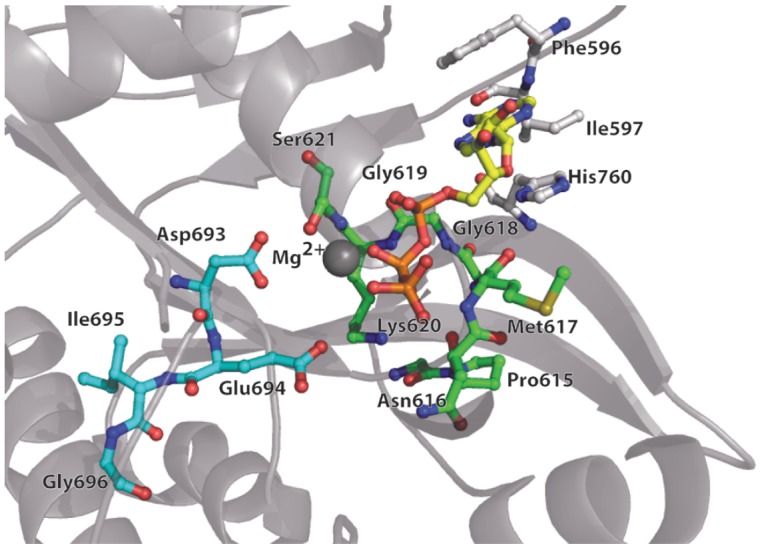
MutS protein ATPase domain. ADP-contacting amino acid residues are shown in light-gray; amino acids of the Walker motif from subunit 1 are indicated in green; from subunit 2 – in cyan; the ADP molecule – in yelloworange; and Mg^2+^ – in gray

The MutS protein belongs to ABC-family ATPases (ATP binding cassette). The proteins of this class, such as membrane transferases, bind to the substrate and hydrolyze ATP to regulate their activity. Certain members of this family demonstrate dimerization of the ATPase domains [96, 97]. The area of dimerization in the region of the ATPase domains in the MutS protein is significant and equal to 2922 A^°2^ [85]. A characteristic feature of the proteins from ABC-family ATPases is a conservative loop protruding from one subunit and complementing the active site of the ATPase domain in another subunit. The binding of ATP or ADP occurs in a classic way characteristic of ATPases through the P-loop (phosphate-binding). The position of the adenine base is fixed from two sides by the aa His760 and Phe596 (in MutS from *E. coli*, *Fig. 4*). The conserved Ile597 forms two hydrogen bonds with the nucleotide. Ser621 coordinates complex formation consisting of a Mg^2+^ ion and β-phosphate of ADP with the involvement of the four water molecules [55]. The Walker motif (D-E-X-X, where X is any amino acid) in MutS from* E. coli *formed by the aa 693–696 stabilizes the water molecules associated with Mg^2+^ [55]. Substitutions of these aa result in loss of the ATPase activity of MutS and inactivation of the repair system [85].

The data obtained using biochemical methods are indicative of significant conformational rearrangements in the ATPase domains upon binding to ATP or its non-hydrolyzable analogs [98-100]. However, only certain aa (Ser668, Asn616 and His728) change their position in relation to the complex in the presence of ADP in the crystal of the MutS-DNA complex in which MutS is bound to two molecules of ATP [85]. According to biochemical data, the affinity of the two ATPase domains for each other is higher upon binding to ATP and lower in the presence of ADP. The general structure of the MutS protein is most compact upon binding to two molecules of ATP, whereas the most relaxed form is observed in the presence of ADP. The ATP- and ADPfree protein has an intermediate conformation [101]. Indirect observations also suggest modulation of protein conformation by nucleotides. For instance, the limited proteolysis patterns of MutS (from *E. coli*) in the presence of ADP, ATP and ATPγS are different from the latter in the absence of nucleotides [98, 99].

In addition, nonequivalence of the two domains of the protein upon binding to ADP (which is characteristic of ABC-family ATPases) was observed in MutS [55, 85]. ADP binds more efficiently to subunit 1 forming specific contacts with a mismatch. Asymmetry of domains is observed even in the absence of DNA [83, 102].

Structural and biochemical data suggest that the conformational changes in the ATPase domain stimulate the rearrangements in the DNA binding domains and vice versa. Transduction of a signal to a distance of ~60 A^°^ and its amplification occurs through the α-helices connecting the two functional domains of the protein and the highly conserved mobile loops of the ATPase domains (*Fig. 4*). It is believed that Glu38, Glu694, Asp693, Asn616, His728 and Ser668 are the key amino acids involved in the signal transduction between the DNA-binding and ATPase domains [84]. Substitution of these aa results in loss of communication between the DNA-binding and ATPase functions of MutS, whereby the protein loses its function in the MMR.

The full-sized MutS protein forms tetramers and oligomers of higher order in solution. MutS tetramerization is important for the suppression of homologous recombination and repair of adducts of cisplatin with DNA [103]. It should be noted that the MutS tetramer is not simply a dimer of dimers as it can bind only one heteroduplex [73]. All crystal structures where MutS was a dimer were obtained using mutants lacking the ability for tetramerization (without C-terminal amino acids 53 aa in the MutS from *E. coli*).

## The stages of MutS protein function in the MMR process

**Fig. 5:**
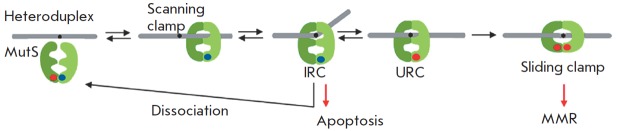
Dynamic model of mismatch recognition by the MutS protein. Heteroduplex is a DNA duplex with a non-canonical pair, modified after [13]

Several stages can be identified in the functioning of the MutS protein (*Fig. 5*). The protein binds nonspecifically to DNA and bends it in a search of a mismatch. Translocation of MutS along the DNA at this stage occurs during linear diffusion [104]. Specific binding to a non-canonical pair of nucleotides leads to confor mational rearrangements in the DNA and the protein with the formation of the initial recognition complex, IRC [13]. Within this complex, the DNA is bent by 60° [55]. Currently, only the crystal structure of this type of complexes with DNA has been established by XRD. The formation of an ultimate recognition complex, URC , has been proposed. In this complex the DNA is straightened and the non-canonical pair of nucleotides is located outside of the double helix. This assumption is based on analogy with other proteins, such as DNA methyltransferases, Tn10 transposase, etc., which, similarly to MutS, “wedge” recognizing amino acids into the DNA from the minor groove side [105, 103]. The protein bound to ATP forms an active conformation of a sliding clamp capable of activating the subsequent stages of mismatch repair.

## The role of the ATPase cycle of MutS

Binding of ADP or ATP to the two subunits of MutS is necessary for the transition from one conformational state to another. It enables the protein to act as a molecular switch [104, 107, 108].

Two nucleotide-binding centers of MutS perform different functions in the MMR [79], which is in accordance with the structural asymmetry established through XRD [55, 78]. Both subunits can simultaneously bind to adenine nucleotides (ATP or ADP) [109]. The dissociation constants for the MutS-ATP or MutS-ADP complex are found in the range of 1–20 μM. Such affinity suggests that the state of MutS wherein one or both of the nucleotide-binding centers are free from nucleotides exists only temporarily. It was demonstrated that MutS exhibits different affinities for ATP, ADP and non-hydrolyzable analogs of ATP. However, there is no unanimity in views regarding the effectiveness of the interaction between these nucleotides and MutS. For instance, even at high concentrations of ADP (100 μM) only one equivalent of a nucleotide per protein dimer would bind to MutS homologues from *E. coli*, yeast, or a human [73, 109, 110]. On the other hand, the ratio of ADP- to ATP-bound nucleotides in MutSα in the absence of DNA equals 1.6. Hence, the protein binds nucleotides in various combinations – ATP/ADP or ADP/ ADP, wherein the second combination emerges as a result of the hydrolysis of the ATP molecule from the first combination [13]. Currently, it is well established that MSH6 (and the corresponding subunit 1 of bacterial MutS) binds to ATP with a higher efficiency than MSH2 (subunit 2 of bacterial MutS) [111, 112]. The ATPase activity of all MutS homologues is stimulated by the presence of DNA (both canonical and non-canonical) [113]. However, the data regarding the impact of the non-canonical pair of nucleotides in the DNA on the ATPase activity of MutS are inconsistent. Several studies have described acceleration (approximately a 4-fold increase) of ATP hydrolysis in the presence of DNA containing a mismatch in comparison with a homoduplex [107]. Other studies [114] have demonstrated that DNA containing a mismatch stimulates the ATPase activity of MutS to a lesser extent in comparison to the DNA with a canonical structure. Both homo- and heteroduplexes accelerate the exchange of nucleotides in the ATPase domains [113]. However, only in the case of a heteroduplex does the cycle of hydrolysis of ATP itself and not the exchange of nucleotides (occurring after hydrolysis) become the rate-limiting step [107].

Coordination of DNA binding and the hydrolysis of ATP processes in the ATPase domains of both subunits of MutS can be described using two schemes. According to scheme 1 [83], the ATPase domain of subunit 1 contains a single molecule of ADP during scanning of the DNA by the MutS protein in search for a mismatch. If the DNA is a substrate of the MMR system, e.g. contains a G/T-pair, MutS forms a specific complex. In this case, the ADP is replaced with ATP in the ATPase domains. The ATPase domain of the second subunit also binds to ATP; the conformational changes then occur in the MutS leading to the formation of a sliding clamp structure. This sliding clamp serves as a signal and recruits the MutL protein which activates the subsequent stages of the repair process. Thereafter, dissociation of MutS from the DNA-containing complex and ATP hydrolysis occur. The MutS protein retains the bound ADP molecule in one of the ATPase domains after completion of the cycle and is ready for a new interaction with the DNA.

Scheme 2 [115] suggests a different approach to the understanding of the nucleotide-binding and ATPase functions of MutS. This scheme is based on XRD data supplemented by calculations using the normal-mode analysis. According to the developed model, subunit 1 binds to and immediately hydrolyzes ATP in the process of the scanning of DNA. ADP release is the ratelimiting step of the ATPase cycle. At this point, only ADP is located in subunit 2. After the formation of a specific complex with a mismatch, both subunits lose their affinity for ADP, then they bind and retain the ATP. Hydrolysis of ATP within the two subunits occurs only after the transition of MutS from the structure sliding clamp into the DNA scanning mode.

In our opinion, the schemes 1 and 2 have significant differences:

1. According to scheme 1 ATP and ADP are absent in subunit 2 during the process of DNA scanning, whereas scheme 2 suggests that subunit 2 at this stage has higher affinity for ADP.

2. According to scheme 1 during DNA mismatch scanning MutS does not hydrolyze ATP; hydrolysis occurs only during the release of MutS from the DNAcontaining complex, while according to scheme 2 the hydrolysis of ATP occurs at the stage of DNA scanning and after the formation of a specific complex.

It can be concluded that there is no clear understanding of the function of the ATPase domains of MutS and of the coordination of their functions at the different stages of the MutS protein action. Hence, the debate over this topic continues.

**Table 3:** Crystal structures of the MutL protein

Organism	Protein fragment	Cofactors and their analogs	Resolution, A^°2^	PDB code	Reference
*Е. coli*	N-terminal domain – ATPase domain fragment (LN40)	-	2.90	1BKN	[122]
	«	ADP, Mg^2+^	2.10	1B62	[121]
	«	ADPnP^1^, Mg^2+^	1.90	1B63	[121]
	«	ADPnP^1^, Mg^2+^, Rb^+^	2.40	1NHH	[123]
	«	ADPnP^1^, Mg^2+^, K^+^	2.00	1NHI	[123]
	«	ADPnP^1^, Mg^2+^, Na^+^	2.30	1NHJ	[123]
	C-terminal domain	Na^+^	2.10	1X9Z	[124]
*Bacillus* *subtilis*	C-terminal domain	-	2.50	3GAB	[125]
	«	-	2.00	3KDG	[125]
	«	Zn^2+^	2.26	3KDK	[125]
*Neisseria* *gonorrhoeae*	C-terminal domain	-	2.40	3NCV	[126]
*Saccharomyces* *cerevisiae* (MLH1/PMS1)	C-terminal domains of the heterodimer	-	2.50	4E4W	-
	C-terminal domains of the heterodimer with the N-terminal domain fragment	Zn^2+^	2.69	4FMN	-
	C-terminal domains of the heterodimer with the exonuclease I fragment	Zn^2+^, Mg^2+^	3.04	4FMO	-
Human (MLH1)	N-terminal domain	ATP	2.50	3NA3	-
	C-terminal domain	-	2.16	3RBN	-

^1^ 5’-adenylyl-β,γ-imidodiphosphate

## MutL PROTEIN – MOLECULAR COORDINATOR OF THE MMR

One of the unique features of the mismatch repair process is the distance of the mismatch from the site of the hydrolysis of the daughter strand of DNA (distance approaching 2,000 bp). Therefore, there has to be a clear coordination in space and time of all the proteins involved in the MMR. A central role in coordinating various stages of the MMR is assigned to the MutL protein. MutL receives a signal regarding the detection of a mismatch and directs the excision repair in the daughter strand of the DNA and DNA repair synthesis. Functioning as a coordinator of mismatch repair processes, MutL interacts with MutS and with the majority of the proteins involved in the subsequent stages of the repair process: MutH, UvrD-helicase, polymerase III and polymerase processivity factors – β-clamp (in prokaryotes) or proliferating cell nuclear antigen (PCN A, in eukaryotes), exonuclease ExoI (in prokaryotes) or polymerase Polη (in eukaryotes) [116].

The role of MutL and its eukaryotic homologues is not limited to the MMR process. It was demonstrated that MutL interacts with the proteins participating in processes involving DNA, such as double-stranded DNA break repair, maintenance of the cellular response to DNA damage, apoptosis, meiotic recombination, and somatic hypermutation [116-119]. All this makes MutL the main element in the coordination of DNA damage recognition and the cellular response to damage in one of the available ways: repair, delay in cell division, or apoptosis [116].

MutL (and its eukaryotic homologues) binds nonspecifically to single- and double-stranded DNA [111, 120]. It is assumed that the interaction of MutL with DNA occurs in complex with MutS. Biochemical studies of the MutL protein are complicated. The latter is attributed to its conformational mobility. In addition, its effect can be evaluated only through a change in the function of its protein partners [116].

MutL, similarly to MutS, functions as a dimer: homodimer in *E. coli *and heterodimer in eukaryotes (MutLα = MLH1 and PMS2, MutLβ = MLH1 and PMS1, MutLγ = MLH1 and MLH3). The molecular weight of MutL from *E. coli *is 68 kDa [121]. The structure of a full-length protein has not yet been established; however, crystals of the C-terminal and N-terminal domains have been obtained separately [122-127]. All structures to date for MutL and its homologues are presented in *Table 3*.

**Fig. 6:**
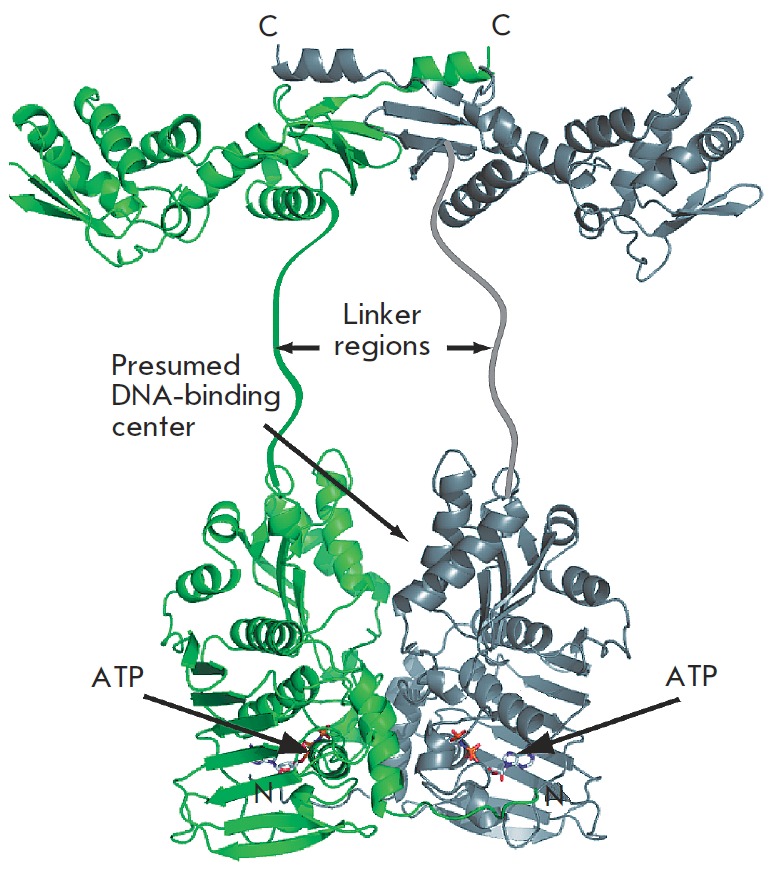
The structural model of a full-length *E. coli *MutL homodimer based on the structures of the N-terminal (PDB code 1B63) and the C-terminal (PDB code 1X9Z) domains

The current model of the MutL structure (*Fig. 6*) was obtained on the basis of XRD data for the N- and Cterminal domains of the protein [128]. According to this model, the N-terminal (aa 1–349) and C-terminal (aa 432–615) domains are interconnected by an unstructured region (aa 350–431) [125, 129]. Interestingly, the primary structure of the C-terminal domain of MutL homologues is less conserved, whereas the secondary structure is conserved. Meanwhile, both the primary and secondary structures of the N-terminal domain are highly conserved.

The C-terminal domains in the MutL dimer are involved in the formation of the primary dimerization interface, and the N-terminal domains contain ATPbinding sites. MutL is an ATPase which belongs to a new family of ATPases containing a novel nucleotide-binding motif. This family also includes topoisomerases of the second type (gyrases), the Hsp90 chaperone protein, and histidine kinases [130]. ATP binding and hydrolysis lead to structural rearrangements in the entire N-terminal domain [122]. The N-terminal domains undergo dimerization in the presence of ADP and ATP. The variable activity of the two ATPase domains of the heterodimers in the ATPase cycle was demonstrated for eukaryotic MutL homologues [131]. The value of the ATPase cycle is significant for the functioning of MutL. Mutant forms of MutL with a lack of the ATPase activity are unable to participate in the repair process and are unable to perform other protein functions [132]. It is believed that ATPase activity is necessary for the MutL protein to modulate protein-protein interactions [122].

Two loops of the MutL positioned in close proximity to the N-terminus are involved in the interaction with MutS, and the groove formed along the lateral surface of the N-terminal domain is involved in the binding to MutH [133] (*Fig. 6*). The saddle-shaped groove located on the surface of the N-terminal domain is most likely involved in the DNA binding. Mutations in the basic amino acids found in this segment, e.g. Arg266, lead to a decrease in the affinity of MutL for DNA and reduce its ATPase activity [134, 135]. However, the assumption regarding the DNA-binding surface in the MutL requires experimental confirmation. Interestingly, MutLα contains an endonuclease motif DQHA(X)_2_E(X)_4_E (where X is any amino acid) which is localized in the PMS2 subunit [136]. This catalytic motif is found in all homologues of MutL, with the exception of some gamma-proteobacteria that are characterized by site-directed hydrolysis of the DNA daughter strand performed by the MutH protein. However, regulation of the catalytic motif of MutL in the hydrolysis of the DNA daughter strand has not yet been confirmed.

## MutH – PROTEIN DIRECTING THE MMR IN E. coli

The MutH protein, a 25-kDa monomeric site-specific nicking enzyme, exhibits similarities to the type II restriction endonuclease Sau3AI [137] and with respect to structure resembles PvuII and EcoRV [138]. The MutH protein binds specifically to a double-stranded sequence 5'-Gm^6^ATC -3'/3'-CT AG↓-5' (location of hydrolysis is indicated by the arrow) and catalyzes the hydrolysis of only one unmethylated, i.e. the newly synthesized DNA strand [16]. Furthermore, MutH also hydrolyzes unmethylated sites, which may cause the emergence of double-stranded breaks [101]. MutH can hardly recognize and hydrolyze a completely methylated DNA sequence [139]. Similar to the majority of type II restriction endonucleases, MutH contains a characteristic motif, Asp-(X)n-Glu-X-Lys (DEK-motif, where X is any amino acid). Two Mg^2+^ ions are required for its catalytic activity [140]. The rate of hydrolysis of the DNA by this enzyme is low; however, it increases significantly in the presence of MutS, MutL, and a DNA mismatch [79]. At low ionic strength of the solution, the activity of MutH is stimulated by the MutL protein without the involvement of the MutS protein bound to a mismatch [91].

**Fig. 7:**
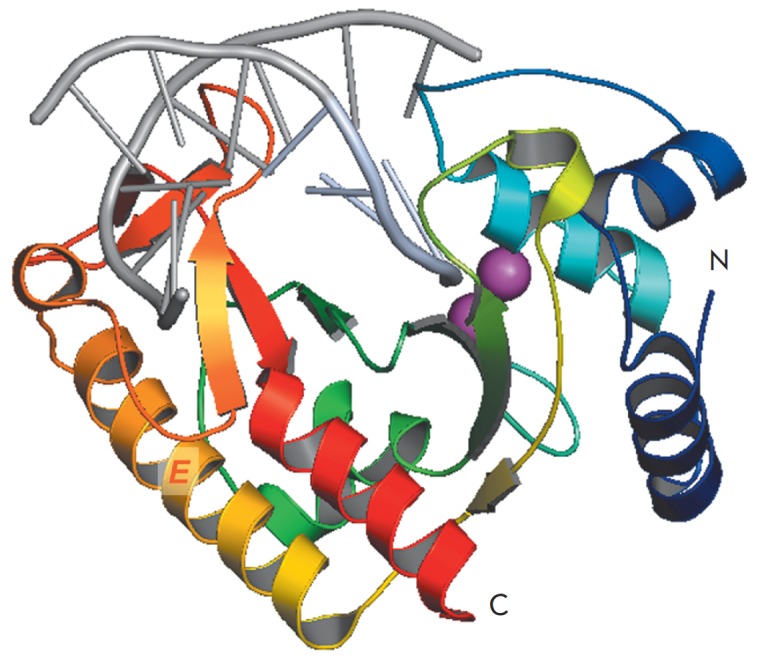
The crystal structure of the *Haemophilus influencia* MutH-DNA complex (colored in gray) containing a hemimethylated 5’-Gm^6^ATC-3’/3’-CTAG-5’ site (PDB code 2AOR). The two Ca^2+^ ions coordinated in the complex are shown in magenta. The *Е *α-helix of MutH interacting with the MutL protein is indicated

The crystal structure of the MutH from *Haemophilus influenzae *(61% similarity with MutH from *E. coli*) in complex with DNA and in the absence of the latter has been determined [137, 140]. With respect to folding, the enzyme resembles the type II restriction endonuclease known as PvuII [138]. The MutH apoenzyme is a clamp consisting of two “arms” (N- and C-“arms”,*Fig. 7*) separated by a large DNA-binding pocket. The catalytic center is located in the N-“arm.” The amino acids responsible for the specific binding to the proteinrecognition site, in particular those that form contacts with heterocyclic bases, are located in the C-“arm.” When specific DNA binding occurs, the protein undergoes compaction, results in a rotation of both “arms” towards each other by an angle of 6-18° in comparison to the closed apoform of the protein, and the DNA-binding pocket becomes narrower tightly covering the recognition site. The structure of the DNA also undergoes restructuring. This includes the unmethylated recognition site becoming more prominently curved and distorted (the bending angle is approximately 30°) in comparison with the hemimethylated site. Nevertheless, local DNA-protein contacts with recognition sites in the two complexes do not differ. However, hemimethylated DNA is more tightly gripped by the enzyme than the unmethylated site (the areas of the DNA-protein contact are 2100 and 1850 A^°2^, respectively). As a result, the DEK-motif interacts with the DNA more efficiently, which leads to a 10-fold increase in the rate of hydrolysis of a hemimethylated recognition site as compared with the unmethylated one [140]. Therefore, in the case of the MutH protein, the bending degree of the DNA does not correlate with the efficiency of its hydrolysis. Single amino acid substitutions in the DNA-binding pocket have revealed that Tyr212 is important in the determination of the methylated status of the DNA [139].

An important feature of MutH is the increase in its catalytic activity during the MMR process. Up to now the mechanism of stimulation of MutH activity remains unclear. The DNA-binding channel in the crystal structure of the MutH protein apoform is not sufficiently wide to bind the DNA. It is assumed that binding of MutL to MutH widens the DNA-binding channel of the latter, increasing the rate of MutH binding to DNA [140]. As was shown using protein-protein crosslinking, MutL interacts with the C-terminal α-helix *E *located on the surface of MutH globule [141] (*Fig. 7*). Perhaps the formation of protein-protein contacts facilitates the rotational movement of the C-“arm” of MutH; as a result, the DNA-binding pocket becomes more accessible for binding to the substrate [132, 140].

## INTERACTION OF MutS, MutL, MutH AND DNA

As was previously mentioned, a ternary complex consisting of MutS and MutL proteins associated with DNA is the key intermediate in the DNA mismatch repair process. It coordinates all stages of the repair after the recognition of a mismatch (i.e. excision repair including DNA unwinding towards the mismatch) and also participates in the transduction of the signal regarding DNA damage to other systems of the cell that control cell division and the triggering of apoptosis [142]. However, the structure of this complex has not yet been elucidated. Furthermore, the MutL protein itself exhibits a relatively low affinity for DNA, particularly for its short linear fragments. Binding to DNA occurs more efficiently in the presence of MutS, Mg^2+^ ions, and ATP [83, 115].

**Fig. 8:**
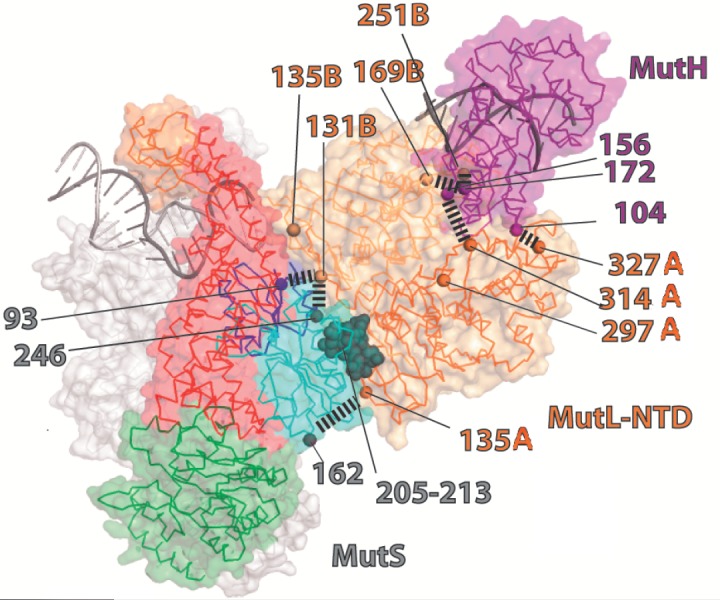
Structural model of the MutS-MutL-MutH-DNA complex. MutS is shown in gray (the mismatch-binding domain is shown in red, the linker domain – in green). N-Terminal domains (NTD) of the two subunits of the MutL dimer are indicated in dark and light orange. The C-terminal and the linker domains of MutL are not shown. The MutH protein is highlighted in purple. The model is based on the following structures: MutS (PDB code 1E3M), MutL (PDB code 1B63), and MutH with DNA (PDB codes 2AZO, 2AOR). The amino acids involved in protein-protein contacts formation are shown in the figure. Colors of numbers indicate the amino acids residues correspondence to definite proteins

The ternary complex (MutS, MutL and DNA) has a dynamic nature; hence, it is impossible to investigate it using the XRD method. In order to investigate the areas of contact between the MutS and MutL proteins, a mutational analysis and hydrogen/deuterium exchange mass spectrometry were used. It was established that aa of MutS, crucial to the formation of contacts with MutL, are located in its connector domain [143]. The N-terminal and ATPase domains of MutL are involved in the interaction with MutS [133]. In addition, detailed studies were conducted based on site-directed protein-protein crosslinking (using bifunctional chemical agents that react with the cysteine residues of the protein) combined with fluorescent methods [144]. Before, crosslinking mutant forms of the MutS and MutL proteins containing a single cysteine residue in a designated position were produced. On experimental data Winkler *et al*. [144] proposed a model of the structure of the complex comprising MutS, MutL, and MutH bound to a mismatchcontaining DNA (*Fig. 8*). In order to build a model of the complex, the authors used a structure of the MutL protein without a C-terminal domain. Previously, it was demonstrated [122] that this domain does not form contacts with the DNA and that the N-terminal domain of MutL is sufficient for the activation of MutH. According to this model, the aa at positions 246 in the MutS and 297 in the MutL (from both protein monomers) are located at a distance of less than 40 A^°^, and the aa 449 in MutS and 297 in MutL are located at a distance exceeding 50 A^°^. This model does not describe all the possible interactions of biopolymers; further investigations are required for a deeper understanding of the processes involved. Furthermore, the model does not account for the previously described [95] transition of the DNA from a bent shape into a linear shape following the activation of MutS that precedes the interaction of MutS with MutL.

The model of a complex consisting of the MutS, MutL, MutH proteins and DNA proposed by Winkler *et al*. [144] is based on their previously published model of the interactions between the proteins MutL and MutH [133]. The distances between the two proteins and interaction surfaces have also been determined using mutant forms of MutL and MutH containing a single cysteine residue, as well as thiosulfate reagents and photo-crosslinkers. It was concluded that the existence of the complex is feasible in which all three molecules, MutS, MutL and MutH, are in close proximity to each other. The formation of a DNA loop separating the proteins is not required in this case, which enables the complex to slide along the DNA in search for a signal of discrimination between the parent and daughter DNA strands.

**Fig. 9:**
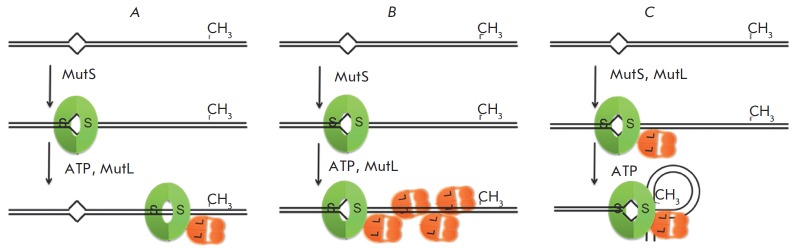
Models of coordination between a mismatch and the hemimethylated 5’-Gm^6^ATC-3’/3’-CTAG-5’ site: cis- (**A** and **B**) and trans- (**C**) models. With respect to another classification: models of a sliding clamp (**A**), multiple MutL polymerization on DNA (**B**) and DNA looping (**C**)

## MODELS OF COORDINATION BETWEEN THE DNA RECOGNITION AND THE CLEAVAGE SITES IN THE MMR SYSTEM

Currently different views exist regarding the processes that occur after the formation of the ultimate recognition complex. A number of articles describe attempts to systematize these models [13, 14, 59, 145]. However, this only complicates the situation as the same phenomena are described using different terminologies and, conversely, the same terms apply to different processes. In the present review we attempted to summarize the existing models of signal transduction from a mismatch to the proteins that perform excision repair basing on the principles of physical interaction of the repair proteins with DNA. The connection between the DNA-binding and the nucleotide-binding functions of the proteins is discussed above and is not considered in order to provide a simplified understanding.

A mismatch and a single-stranded break in *E. coli* cells are separated by significant distances (approaching 2,000 bp) during the stage of signal transduction of the daughter strand detection and subsequent excision repair [146]. The process is bidirectional in nature; i.e., excision occurs in both directions relative to the mismatch [61, 147]. These experimental facts form the basis of all models. Various views regarding the mechanism of initiation of the MMR process are summarized in *Fig. 9*.

Existing trans- and cis-models [13] regarding coordination between the DNA recognition and cleavage sites in the MMR system differ with respect to whether significant conformational rearrangements of the DNA are required (e.g., formation of α-shaped loops) or not, respectively. Examples of the cis-mechanism of action can be found amongst restriction endonucleases (types I and III), and a trans-mechanism can be frequently encountered during the transcriptional regulation of genes [145].

The basis for creating a model also includes another feature – whether MutS (or a MutS–MutL–DNA ternary complex) remains associated with the mismatch or moves away from it. Stationary and sliding clamp models can be distinguished. To date, all of the abovementioned models are supported by experimental evidence. The sliding clamp model is the most popular one [98, 148-150]. According to this model, MutS loses affinity for the mismatch and forms a structure of a unique DNA-clamp in the ultimate recognition complex containing DNA and two molecules of ATP. In this case, the protein dimer has two channels separated by central (mismatch-binding) domains, the larger of which binds to DNA (*Fig. 3*). Significant restructuring occurs within MutS during the formation of a sliding clamp. It is assumed that the central (mismatch-binding) domains from each subunit of the dimer rotated away from each other, and, hence, the channel size in which the DNA is located increases by a factor of 2 as a result of combination of the two channels [44]. However, these assumptions need to be experimentally verified. In the sliding clamp conformation MutS serves as a “turned on” switch capable of translocating along the DNA and activating the functions of other proteins in the MMR system. Hydrolysis of ATP is not required in order for this type of translocation of MutS to occur [104]. “Molecular clamps” perform important functions in the DNA metabolism; e.g., PCN A directs DNA replication and increases the processivity of DNA polymerase. This model is supported by the fact that bacterial MutS proteins and their eukaryotic homologues in the presence of ATP slide away from the DNA fragment containing a non-canonical base pair, and then from the ends of linear DNA (if they are not blocked by bulky groups or tightly bound proteins) [98, 151]. Recent studies [104] carried out employing fluorescence techniques enabled to estimate the lifetime of a sliding clamp. It was found to be relatively long and was approximately 10 min. The discussed mechanism suggests the possibility of the binding of several molecules of MutS to DNA containing a mismatch, which can improve the efficiency of a repair process [145].

According to other models, MutS must remain bound to the DNA. For instance, Kunkel and Erie [13] suggest that the ATP-dependent translocation of MutS away from the mismatch is not necessary for its functioning, and only conformational changes in the protein are important for the subsequent repair events to occur. This model is supported by the fact that the lifetime of the MutS–MutL–DNA ternary complex bound to ATP in the region containing a mismatch is longer than that for individual MutS molecules activated by a mismatch and bound to ATP [145, 151, 152]. It is highly probable that *in vivo *MutS can translocate away from the mismatch but only for short distances as the results of footprinting [152] and studies performed using the surface plasmon resonance technique [150] demonstrate that the DNA site in the mismatch region is covered by bound proteins. Kunkel and Erie also suggest that DNA bending in the mismatch-containing region or any DNA deformation caused by the MutS protein must be maintained during all phases of the MMR, which will serve as a directing and probably terminating signal during exonuclease degradation of the DNA daughter strand [13]. This is only feasible if the contact between the mismatch and the MutS is preserved.

According to another stationary model, the signal transduction from MutS to MutH (between the mismatch site and the strand discrimination site) occurs as a result of a large number of MutL molecules binding to the DNA (formation of nucleoprotein filaments) until the strand discrimination site is reached (*Fig. 9B*) [153]. Experimental confirmation of this model has been recently obtained. The fluorescence microscopy technique was used on live cells producing fluorescently labeled MutL and MutS proteins; it was demonstrated that in the mismatch region the number of MutL protein molecules exceeds that of MutS by a factor of 3 [154]. However, this number is not sufficiently large to be able to unambiguously confirm the model of polymerization.

There are models suggesting DNA looping out (transmodels,* Fig. 9C*). The first suggestion of such a mechanism was proposed as a result of an investigation of the MutH activation in the presence of MutS and MutL. In the experiment the mismatch was located on one plasmid and the 5'-Gm^6^ATC -3'/3'-CT AG-5' site – on another. In the control group, both sites were located on the same plasmid. DNA cleavage efficiency in both cases coincided [152]. Moreover, protein-free DNA is rarely encountered within the cell. Typically, almost immediately after replication it becomes structured with the involvement of proteins and as a result MutS sliding along the DNA is hindered [115]. The data obtained using atomic force microscopy also support the model that includes looping out of DNA. These data indicate the importance of MutS tetramerization in the presence of ATP [148, 155]. Two types of MutS-DNA complexes can be identified in the microphotographs. The first type consists of a MutS-DNA dimer, and the other is a DNA loop formed by two protein dimers. Hence, MutS homodimers can be assigned to two groups with respect to the functions where a certain number of molecules remain bound to the mismatch and the other pull the DNA through itself, maintaining contact with the first dimer. The “immobile” group of MutS dimers can result from the hydrolysis of ATP in one of the domains of the dimer. Both the cis- and trans-mechanisms of the MMR process can be explained from the point of view of this “combined” mechanism.

## CONCLUSION

Currently, various views regarding the MMR mechanism exist; therefore, extensive ongoing research in this area still continues. The identification of a single mismatch amongst many thousands of canonical base pairs in the DNA is a unique process [155]. The fluorescence resonance energy transfer technique at the single molecule level has enabled to identify many conformations of the MutS protein in the presence of canonical DNA ligands [104]. However, binding of the MutS protein to DNA during the search for a mismatch, which is a key event of the MMR process, has not yet been fully characterized. The pending issues concern not only the short-lived intermediate MutS-DNA complexes but more complicated complexes as well: MutS–MutL– DNA and MutS–MutL–MutH–DNA. In order to characterize these complexes, one can use a combination of various optical [95, 153, 154] and fluorescence [104] techniques associated with crosslinking of proteins to proteins and proteins to DNA [144]. A recently proposed approach to investigating short-lived complexes based on the covalent fixation of MutS to the DNA is considered to be rather promising [156].

Investigations of the MutS structure during DNA scanning are required. It is believed that the mismatchbinding domains of both subunits of the MutS dimer lose affinity for each other: thereby, the protein channel in which the DNA is located undergoes a 2-fold increase in size [44]. The study of mutual coordination of MMR system proteins is a particularly complicated issue. The same is true for the influences of other cellular proteins on the activity of the abovementioned proteins. It is obvious that further research is required to create a complete picture of the MMR repair system functioning in living cells.

## Abbreviations

aa: amino acid residue
bp: base pair
HNPCC: hereditary nonpolyposis colon cancer (Lynch syndrome)
HTH: helix-turn-helix
IDL: insertion-deletion loop
IRC: initial recognition complex
m6A: N6-methyl-2'-deoxyadenosine
MMR: mismatch repair
PCNA: proliferating cell nuclear antigen
SSB protein: single-strand binding protein
URC: ultimate recognition complex
XRD: X-ray diffraction analysis
